# Low-frequency whole-body vibration can enhance cartilage degradation with slight changes in subchondral bone in mice with knee osteoarthritis and does not have any morphologic effect on normal joints

**DOI:** 10.1371/journal.pone.0270074

**Published:** 2023-08-17

**Authors:** Haiming Wang, Chi Zhang, Siyi Zhu, Chengfei Gao, Qiang Gao, Ridong Huang, Sijia Liu, Xiangyang Wei, Huakai Zhang, Quan Wei, Chengqi He

**Affiliations:** 1 Rehabilitation Medicine Department, The First Affiliated Hospital of Zhengzhou University, Zhengzhou, Henan, China; 2 Center of Rehabilitation Engineering Technology Research, Henan Province, Zhengzhou, Henan, China; 3 Rehabilitation Medicine Department, The Affiliated Hospital Of Southwest Medical University, Luzhou, Sichuan, China; 4 Department of Rehabilitation Medicine, Southwest Medical University, Luzhou, Sichuan, China; 5 Nuclear Medicine and Molecular Imaging Key Laboratory of Sichuan Province, Luzhou, Sichuan, China; 6 Rehabilitation Medical Center, West China Hospital, Sichuan University, Chengdu, Sichuan, China; 7 Rehabilitation Medicine Key Laboratory of Sichuan Province, Sichuan University, Chengdu, Sichuan, China; 8 Rehabilitation Medicine Department, The Affiliated Hospital of Qingdao University, Qingdao, Shandong, China; 9 Department of Biomedical Engineering, The Hong Kong Polytechnic University, Hong Kong, China; 10 Department of Respiratory and Critical Care Medicine, Targeted Tracer Research and Development Laboratory, West China Hospital, Sichuan University, Chengdu, Sichuan, China; 11 Medical College of Zhengzhou University of Industrial technology, Zhengzhou, Henan, China; Drexel University, UNITED STATES

## Abstract

**Purposes:**

To evaluate the effects of low frequency whole-body vibration (WBV) on degeneration of articular cartilage and subchondral bone in mice with destabilization of the medial meniscus (DMM)induced osteoarthritis(OA) and mice with normal knee.

**Methods:**

Ten-week-old C57BL/6J male mice received DMM on right knees, while the left knees performed sham operation. There were six groups: DMM, SHAM DMM, DMM+WBV,SHAM DMM+WBV, DMM+ NON-WBV and SHAM DMM+NON-WBV. After four weeks, the knees were harvested from the DMM and SHAM DMM group. The remaining groups were treated with WBV (10 Hz) or NON-WBV. Four weeks later, the knees were harvested. Genes, containing Aggrecan(Acan) and CollagenⅡ(Col2a1), Matrix Metalloproteinases 3 and 13(MMP3,13), TNFα and IL6, were measured and staining was also performed. OA was graded with OARSI scores, and tibial plateaubone volume to tissue volume ratio(BV/TV), bone surface area to bone volume ratio (BS/BV), trabecular number(Tb.N) and trabecular thickness separation(TS) between groups were analyzed.

**Results:**

Increased OARSI scores and cartilage degradation were observed after WBV. BV/TV, Tb.N and TS were not significant between the groups. Significant reductions were observed in MMP3, MMP13, Col2a1, Acan, TNFα and IL6 in the DMM+WBV compared to SHAM DMM+WBV group. BV/TV, BS/BV, Tb.N, TS and OARSI scores were not significantly changed in the left knees. IL6 expression in the SHAM DMM+WBV group was significantly increased compared with the SHAM DMM+ NON-WBV group, while Col2a1, Acan and MMP13 expression decreased.

**Conclusion:**

WBV accelerated cartilage degeneration and caused slight changes in subchondral bone in a DMM-induced OA model. WBV had no morphologic effect on normal joints.

## Introduction

Osteoarthritis (OA) [1], the most common form of arthritis worldwide, is characterized by pain, stiffness, swelling, and weakness of function, and is a leading cause of disability in the aged population, affecting any synovial joint, such as knee, hip, shoulder and hand. With increasing age and body weight, the prevalence of OA increases, causing a major burden on society [2]. Athough OA is caused by biological [3] and biomechanical factors [4], the specific pathogenic mechanism is still unclear and involves loss of cartilage and sclerosis of subchondral bone [5]. Due to the lack of curative treatment for OA, comprehensive therapies, including self-management and education, exercise, and weight loss, are employed to delay invasive surgical methods, such as joint replacement [1, 6].

Regulation of mechanical loading is central to the development of OA [7]. Many studies have previously demonstrated that decreased muscle strength around the knee joint is an important factor in the occurrence of knee OA [8], and strengthening the knee joint can prevent the progression of this disease [4, 9]. Therefore, biomechanical interventions and strength training are appropriate treatment modalities for all individuals with knee OA, as recommended by researchers [10] and the recent guidelines of the OA Research Society International (OARSI) [11, 12] and Academy of Orthopedic Surgeons (AAOS) [13, 14].

Whole-body vibration (WBV) is a popular exercise modality in sports, fitness [15] and physiotherapy, causing a specific myotatic reflex to induce muscle contraction and develop muscle strength and power [16–18]. This technique affects, the nervous system [19], peripheral circulation [20] and other systems [16]. In terms of OA, multiple studies [10, 21, 22] and some meta analysis researches [17, 23], including our previous research [24, 25], verified that WBV could improve the function and increase the strength, of patients without causing any adverse effects. However, other researchers [26–28] found no additional effect of WBV, compared with conventional exercise, and a meta analysis reported similar outcomes [29]. The guidelines [12, 14] do not recommend and also are not against WBV for treatment of OA, probably because of these controversial results. Similarly, many scholars [30–32] firmly believe that WBV is an effective training method and a potentially feasible intervention for OA management in the future.

Different studies employed different parameters (including frequency, amplitude, magnitude, and durations) for WBV training, which maybe responsible for the varying conclusions. Frequency is one critical factor for WBV, and the range required by IOS2631-1 is from 0.5 Hz to 80 Hz for health and comfort [33]. In fact, the frequency most commonly used clinically ranged from 5 to 45Hz [15, 24]. McCann MR [34, 35] found that WBV (45 Hz) could degrade the cartilage of CD-1 normal mice, while C57BL/6 mice showed resistance to cartilage degeneration. Pamon T [36] revealed that exposure to 90Hz WBV training could improve cartilage thickness in obese mice. These results showed that WBV at different frequencies produced different effects on normal animals with different genotypes. Our latest study revealed that 5 Hz WBV could delay cartilage degeneration and preserve subchondral bone microarchitecture in a mouse model of OA [37]. Junbo W [38] reported that 10 Hz and 20 Hz WBV treatment relieved pain and improved cartilage formation in a rabbit model of OA, while 30 Hz and 40 Hz had the opposite effects. Qin J [39] also revealed that 35 Hz vibration accelerated cartilage degeneration in a rat model of OA. The literature clearly shows different outcomes between animal studies and clinical studies. However, to the best of our knowledge, most studies indicated that WBV had beneficial effects on function and muscle strength, and there were no clinical side effects (such as muscle soreness, muscle fatigue or dizziness) reported in WBV therapy for OA [21, 24, 29, 40]. Most importantly, researchers cannot directly assess joint structure in the clinical via histological staining after patients with OA undergo WBV. Additionally, studies of WBV with a low frequency (10 Hz) in a mouse model of OA are lacking.

How does WBV work in individuals with OA? Are there no side effects, or are there potential side effects that have not been observed? In addition, can WBV safely be used for fitness in a healthy population, weight loss, and other nonarthritic patients due to its effects on knee joints? Therefore, we performed this further research about WBV, to explore a low frequency WBV (10 Hz) could improve or degrade joint cartilage in an OA mouse model and whether have side effects (cartilage or/and subchonral bone degradation) on normal joints or not.

## Materials and methods

### Animals and experimental design

All procedures were approved by the Animal Ethics Committees of West China Hospital, Sichuan University and performed according to the Guide for the Care and Use of Laboratory Animals. All C57BL/6Jmale mice (n = 36) were obtained from the Jackson Laboratory. The mice were housed in groups of three/cage in ventilated racks with specific pathogen-free barrier conditions at a constant temperature (22°C) with a 12-hour light/darkness cycle and, normal food and water provided *ad libitum*. When the mice were 10 weeks old, they were randomly divided into three groups: the baseline group (n = 12), the WBV group (n = 12)and the control group with NON-WBV(n = 12). The right knees of each group of mice underwent destabilization with medial meniscus (DMM) surgery (mice were anesthetized by injection of 50mg/kg sodium pentobarbital), which was recommended by the OARSI [41, 42], while the left knees underwent a sham operation performed by making a shin incision at the same location. Mice were placed on a warming pad after surgery until fully awake and given buprenorphine for pain relief (0.05 mg/kg, twice a day for three days). Penicillin was injected intraperitoneally for 3 days (50,000 U/kg/d). There were six groups: the DMM group (right knees of the baseline group, n = 12), the SHAM DMM group (left knees of the baseline group, n = 12), the DMM+WBV group (right knees of the WBV group, n = 12), the SHAM DMM+WBV group (left knees of he WBV group, n = 12), the DMM+ NON-WBV group (right knees of the mice subjected to NON-WBV, n = 12) and the SHAM DMM+ NON-WBV group (left knees of the mice subjected to NON-WBV, n = 12). Four weeks later, knees were harvested from 12 mice in the baseline group. The remaining groups were treated with WBV or NON-WBV. The baseline group and the other groups were weighed weekly until mice were sacrificed under anesthesia (sodium pentobarbital, 50mg/kg) via cervical dislocation.

### WBV

Four weeks after the surgery, WBV was provided by a vertical sinusoidal vibration platform (frequency 10 Hz, peak to peak amplitude 4 mm, gravitational acceleration 0.73 g, i-vib5050A; BodyGreen, Changhua County, Taiwan) for 20 min/day, 5 days/week for 4 weeks. During treatment the mice were housed in cages without a bottom on the vibration platform, while the mice that received the NON-WBV were placed on the same inactive platform with the same regime. Following WBV, the mice were returned to conventional housing and monitored daily. The mice were euthanized by cervical dislocation to obtain knee joint tissues, 24 h after the final exposure to WBV.

### Analysis of the knee joint diameters

The knee joint samples were harvested and dissected free from all soft tissue (muscles and ligaments) to obtain the distal femur and proximal tibia, with the complete capsule retained. Then the maximum diameter of the knee joint was immediately measured via a Vernier caliper, by three different researchers for three times and the mean value was calculated.

### Micro-computed tomography (micro-CT)

Six knees of each group were isolated, fixed in 4% paraformaldehyde for 24 h and then scanned on an Inveon Multi-Modality micro-CT (Siemens), at an energy 80kV and intensity of 500 μA, based on protocols in our previous study [43]. Inveon Research Workplace, version 4.0 was used for three dimensional (3D) reconstruction of the whole knee joint. The region of interest (ROI) was defined from the tibial growth plate to the tibial plateau(Fig 3I). Bone microstructure parameters included bone volume to tissue volume ratio (BV/TV), bone surface area to bone volume ratio (BS/BV), trabecular number (Tb. N) and trabecular thickness separation (TS).

### Histological assessment and scoring

Following micro-CT, the limbs were placed in 4% paraformaldehyde solution for 2 days and then decalcified for 12 days in 20% ethylene diamine tetra acetic acid(EDTA) in PBS (PH 7.0), processed, and embedded in paraffin wax, and 5μm coronal sections were obtained following the OARSI [44]. Serial sections were harvested every 75μm to encompass all weight-bearing areas of the knee joint. The sections were stained with 0.1% Safranin O/0.02% fast green to detect proteoglycans and glycosaminoglycans. Immunohistochemistry staining for Collagen II (Col2a1) (1:100, bs-10589R; Bioss, Beijing, China), Aggrecan (Acan) (1:200, bs-11655R; Bioss), MMP3(1:50, Ab52915; Abcam, Cambridge, UK), MMP13 (1:200, Ab39012; Abcam), IL6 (1:100, ab290735; Abcam) and TNFα(1:1000, ab307164; Abcam) were conducted on paraffin sections following appropriate antigen retrieval methods. The sections were imaged via an Axio Scope Light Microscope (Zeiss). The tibial plateau quadrants of the knee joint were scored by two independent blinded observers according the mouse recommendations of OARSI [44]. OA severity is expressed by the mean maximum score.

### Gene expression analysis

Six other knee joint specimens from each group were dissected free from all soft tissue (muscles and ligaments) to obtain the distal femur and proximal tibia, which contained cartilage and subchondral bone (the different groups’ samples were stored in -80°C freezer before testing [45]). Total RNA was extracted using TRIzol reagent, according to protocol provided by the manufacturer (Invitrogen). A total of 1 μg RNA was reverse transcribed to cDNA using the PrimeScript RT reagent kit (TaKaRaBio). Real-time quantitative PCR was performed with 2 μL of cDNA with the SYBR Premix Ex Taq II kit (TaKaRa Bio) in CFX Real-Time PCR Detection System (Bio-Rad). The relative mRNA expression levels were normalized to those of GAPDH in the same sample and analyzed with the 2^-ΔΔCT^ method as our previous report [43]. Genes involved with cartilage anabolism were: Aggrecan (Acan) and Collagen II (Col2a1), those involved in cartilage catabolism, such as Matrix Metalloproteinases 3 and 13 (MMP3,13), and the joint inflammation-related genes TNFα and IL6 were measured. The sequences of the oligonucleotide primers are listed in Table 1.

**Table 1:** Oligonucleotide primers for RT-PCR.

Gene	Forward Primer	Reverse Primer
*Gapdh*	GTCGTACCACAGGCATTGTGATGG	GCAATGCCTGGGTACATGGT GG
*Mmp3*	GGCCTGGAACAGTCTTGGC	TGTCCATCGTTCATCATCGTCA
*Mmp13*	TGTTTGCAGAGCACTACTTGAA	CAGTCACCTCTAAGCCAAAGAAA
*Acan*	CTGGGATCTACCGCTGTGAAG	GTGTGGAAATAGCTCTGTAGTGGAA
*Col2A1*	TGGTGGAGCAGCAAGAGCAA	CAGTGGACAGTAGACGGAGGAAAG
*TNFα*	CCCTCACACTCAGATCATCTTCT	GCTACGACGTGGGCTACAG
*IL6*	TAGTCCTTCCTACCCCAATTTCC	TTGGTCCTTAGCCACTCCTTC

### Statistical analyses

All values were analyzed using SPSS 22.0 software and are shown as the means ± SD. Statistically significant differences were assessed by one-way analysis of variance (ANOVA) followed by Tukey’s post hoc analysis test for comparison of different groups. A *P* value of <0.05 was considered significant.

## Results

### Diameter changes of the knee joint

To evaluate the effects of low-frequency WBV on knee OA and assess whether the WBV protocols used clinically induced side effects, we exposed male C57BL/6 mouse that underwent the OA procedure to vertical sinusoidal WBV (20 min/day, 5 days/week, at 10 Hz, 0.73 g peak acceleration)for 4 weeks.

**Fig 1:**
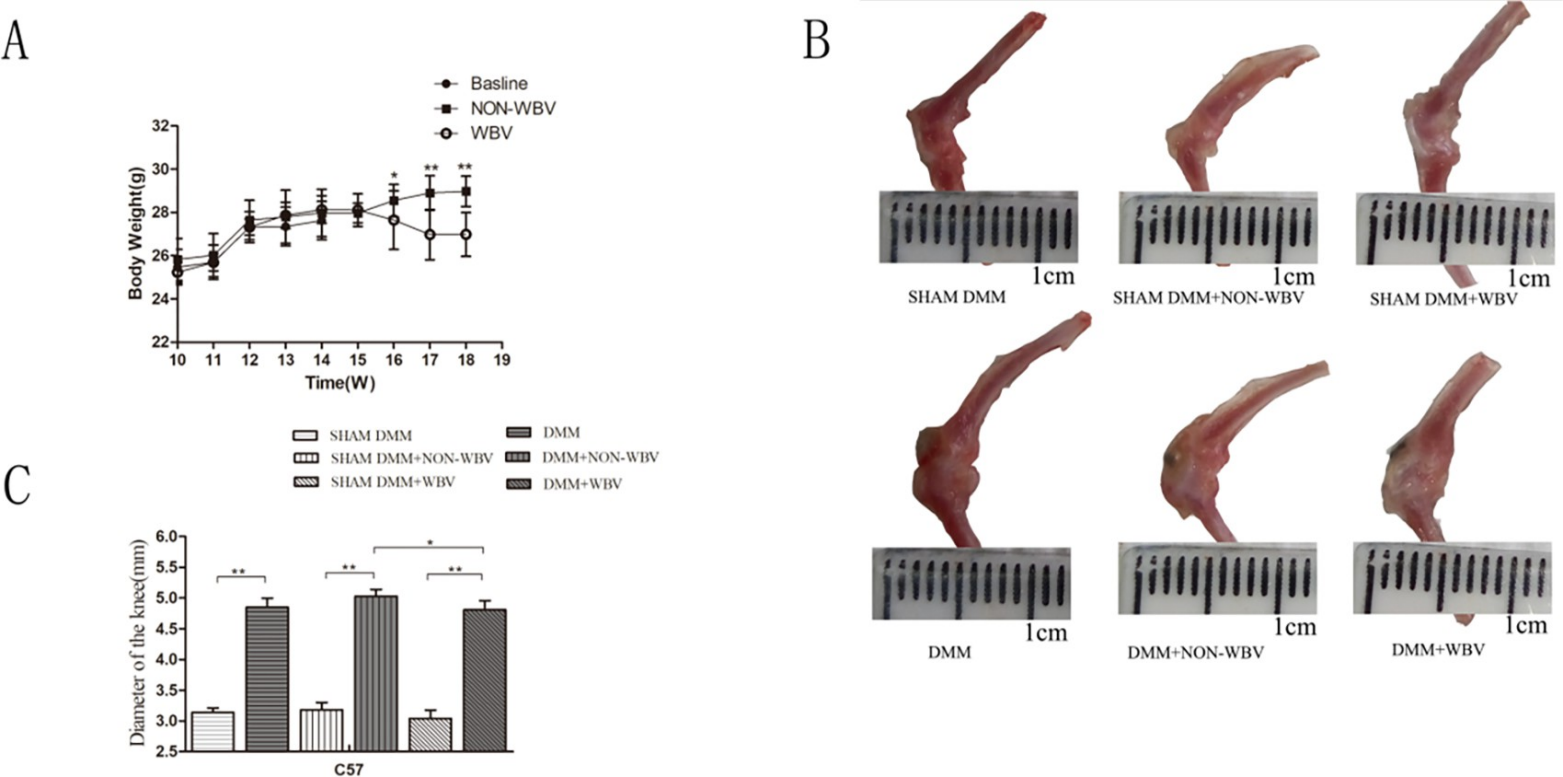
Analysis of the changes in diameter of knee joint following WBV. (**A**) WBV induced weight loss in the mouse model of OA and no weight changes in the normal mice (n = 12). (**B**) Representative knee joint samples with the complete capsule retained, dissected free from muscles and ligaments. (**C**) Joint hypertrophy in the groups underwent DMM surgery than the sham operation groups (n = 6). Data are expressed as the mean ± SD. *P<0.05,**P<0.01.

No significant differences in body weight were found in baseline group, WBV group and the NON-WBV group at the time points tested in the mice 14 weeks of age to 18 weeks of age. WBV group had a significantly decreased body weight from 16 weeks to the end point at 18 weeks (*P* = 0.0273 for comparing NON-WBV DMM in 16 weeks, *P*<0.0001 for comparing NON-WBV DMM in 17 weeks, *P*<0.0001, for comparing NON-WBV DMM in 18 weeks), suggesting that the WBV could result in weight loss in the OA model (Fig 1A).

Joint hypertrophy is one of most important physical signs of OA [46, 47]. We examined the knee joint diameters of the mice via Vernier caliper, and found an obvious increase in the groups that underwent DMM surgery compared with the sham operation groups (*P*<0.0001 for comparing DMM and SHAM DMM, *P*<0.0001 for comparing NON-WBV DMM and SHAM DMM+NON-WBV, *P*<0.0001 for comparing DMM+WBV and SHAM DMM+WBV, respectively). WBV did not improve or aggravate this change in the mice with knee OA. However, the hypertrophic joints of the mice with OA exposed to WBV tended to show alleviation with a significant difference compared with the NON-WBV group (*P* = 0.0285). The knee joint diameters had no changes in the groups with the sham operation, regardless of the WBV treatment(Fig 1B and 1C).

### WBV enhanced cartilage degradation

**Fig 2:**
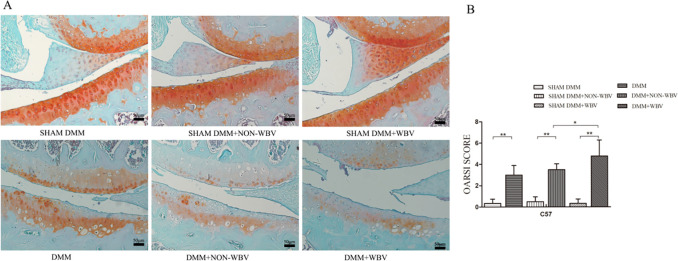
Histologic appearance of the mouse joints after exposure to WBV. (**A**) Representative coronal sections of the knee joint, stained with Safranin O/fast green from the mice, exposed to WBV for 4 weeks and the untreated sham controls. Images are oriented with the femoral condyle on top, and the tibial plateau on the bottom. Damage to cartilage was detected within the joint in all the mice that underwent DMM surgery, regardless of whether they were exposed to 4 weeks of WBV. The mouse model of OA exposed to WBV showed more severely deteriorated cartilage than the OA model with sham WBV. No obvious damage was detected in the control mice with sham DMM. (**B**) Knee joints were scored using the OARSI scoring system to quantify the degree of joint degeneration, and the mean maximum OARSI scores are presented corresponding to the medial articular surface. The mice exposed to 4 weeks of WBV had obvious degeneration, compared with the mice in the sham DMM group and DMM group that were age-matched and not treated with WBV.(n = 6). Data are expressed as the mean ± SD.*P<0.05,**P<0.01.

To assess the changes in cartilage of the knee joint, we scored Safranin O/fast green- stained histological sections. Articular cartilage damage was observed in the groups that underwent DMM surgery compared with the sham operation group (*P*<0.0001 for comparing DMM and SHAM DMM, *P*<0.0001 for comparing NON-WBV DMM and SHAM DMM+NON-WBV, *P*<0.0001 for comparing DMM+WBV and SHAM DMM+WBV, respectively) (Fig 2B). WBV accelerated cartilage degradation in the mice experimental group compared with the mice with the sham vibration (*P* = 0.0408). Joint damage was characterized by loss of glycosaminoglycans in the superficial layer of articular cartilage adjacent to focal defects and focal articular cartilage lesions (Fig 2A). No significant differences were found in the mean OARSI score between the DMM+NON-WBV group and the DMM group (*P* = 0.2700) (Fig 2B)(see Table S1 in S1 File). Of note however, there was no obvious joint damage found in any matched sham operation groups regardless of exposure to WBV. These results showed that WBV could enhance cartilage degeneration and had no influence on the normal knee joint.

### WBV did not induce obvious changes in subchondral bone

**Fig 3:**
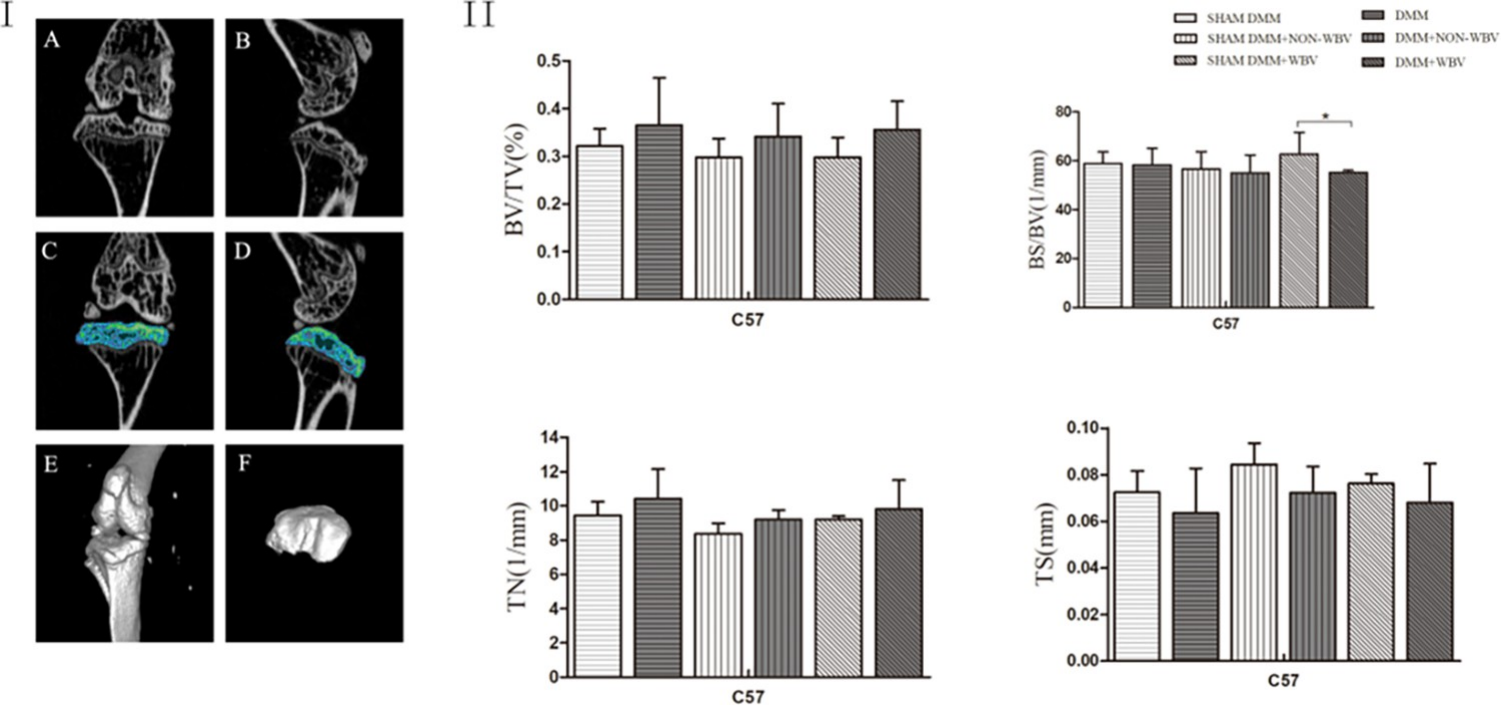
Analysis of tibial subchondral bone microarchitecture after WBV. (**I**) Representative coronal and sagittal micro-CT images of the knee joint (A, B). ROI (the green region) defined from the tibial growth plate to the tibial plateau (C,D).Three-dimensional reconstruction of the whole knee joint (E, F). (**II**) Morphometric analysis of the subchondral bone in all groups, with or without WBV, shows no significant change in the bone microstructure parameters, including bone volume to tissue volume ratio (BV/TV), bone surface area to bone volume ratio (BS/BV), trabecular number (Tb. N) and trabecular thickness separation (TS) in all the groups, except OA group exposed to WBV compared to the matched control group with sham DMM.(n = 6).Data are expressed as the mean ± SD.*P<0.05.

For each sample, the ROI was manually outlined in serial planes to capture trabecular bone within this area. There were no significant differences in the six groups, in the subchondral BV/TV, BS/BV, Tb. N and TS, except the BS/BV in the WBV group compared to the group with the sham operation exposed to WBV (*P* = 0.0472). Notably, exposure to 4 weeks of WBV did not induce a significant change compared to that of the NON-WBV group, but resulted a trend of increased Tb. N and decrease TS(Fig 3II). In addition, within groups, there was also a trend of increase in Tb. N and, BV/TV and a decrease in TS, with no significant differences. (see Table S2 in S1 File).

### Influence of WBV on anabolism, catabolism and inflammation

**Fig 4:**
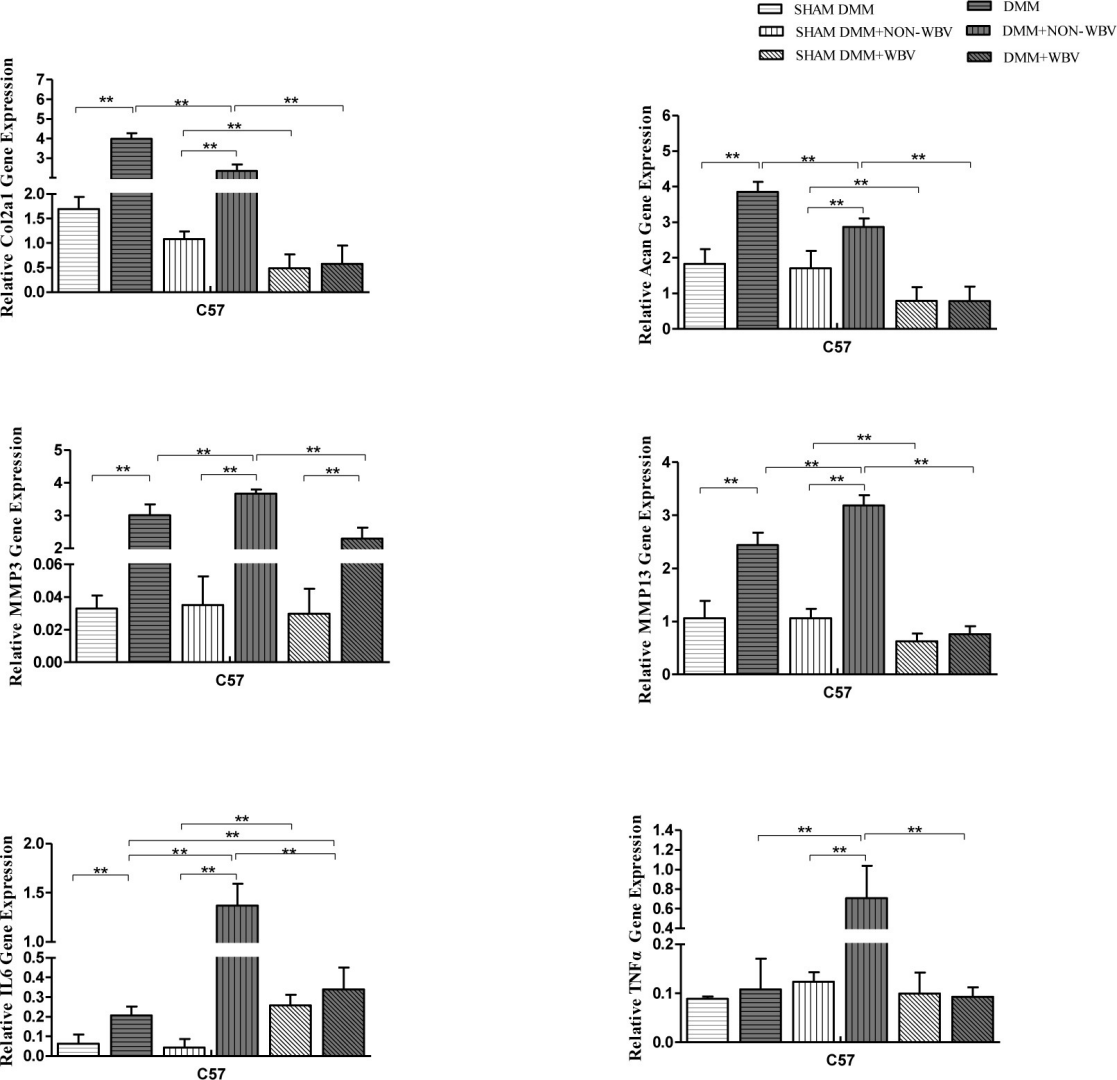
Relative mRNA expression levels of Acan, Col2a1, MMP3, MMP13, TNFα and IL6 in the knee joint cartilage. Compared to the baseline group with sham DMM, the group that underwent DMM surgery exhibited an obvious increase in Acan, Col2a1, MMP3 and MMP13. The mouse model of OA eight weeks after surgery showed a further increase in MMP3 and MMP13, while Acan and Col2a1 showed a decrease, compared with those of the baseline OA group. Obvious increases in TNFα and IL6 were also presented in the mouse model of eight weeks after surgery compared to those in the matched group four weeks after DMM surgery. All the genes detected had a significant decrease in the group with OA and WBV compared with the non-vibrated group. (n = 6). Data are expressed as the mean ± SD.**P<0.01.

We next examined whether WBV resulted in changes in the anabolism, catabolism and inflammation of the joint cartilage. MMP3, MMP13, Acan, Col2a1 and IL6 increased significantly, compared with those in the sham operation group, four weeks after the surgery in DMM group (*P*<0.0001 for comparing DMM and SHAM DMM about MMP3, MMP13, Acan and Col2a1, *P* = 0.0002 for comparing DMM and SHAM DMM about IL6, respectively), while TNFα had a rising trend without statistical significance (*P* = 0.2333 for comparing DMM and SHAM DMM) (Fig 4). Compared to the SHAM DMM group (*P*<0.0001 for comparing DMM+NON-WBV and SHAM DMM about MMP3, MMP13, TNFα, and IL6, respectively) and the DMM group (*P* = 0.0004 for comparing DMM+NON-WBV and DMM about MMP3, MMP13, TNFα, and IL6, *P* = 0.0001, *P*<0.0001, *P*<0.0001, respectively), the DMM+NON-WBV group showed a further increase in MMP3, MMP13, TNFα, and IL6 eight weeks after the surgery, with significant changes, while Acan and Col2a1 had a decrease compared with those of the DMM group (*P*<0.0001 for comparing DMM+NON-WBV and DMM about Acan and Col2a1, respectively) (Fig 4). When WBV was employed, a significant reduction was detected in MMP3, MMP13, Col2a1 and Acan (*P*<0.0001 for comparing DMM+WBV and SHAM DMM+WBV about MMP3, MMP13, Col2a1 and Acan, respectively), similar to TNFα and IL6 (*P*<0.0001 for comparing DMM+WBV and SHAM DMM+WBV, respectively)(Fig 4). Compared to the SHAM DMM+NON-WBV group, the SHAM DMM+WBV group had a significant decrease in Col2a1, Acan and MMP13 (*P* = 0.0005, *P* = 0.0048, *P* = 0.0005, respectively), and an increase in IL6 (*P*<0.0001), without significant changes in MMP3and TNFα (*P* = 0.2899, *P* = 0.1199, respectively)(Fig 4). (see Table S3 in S1 File).

### Immunohistochemistry analysis

**Fig 5:**
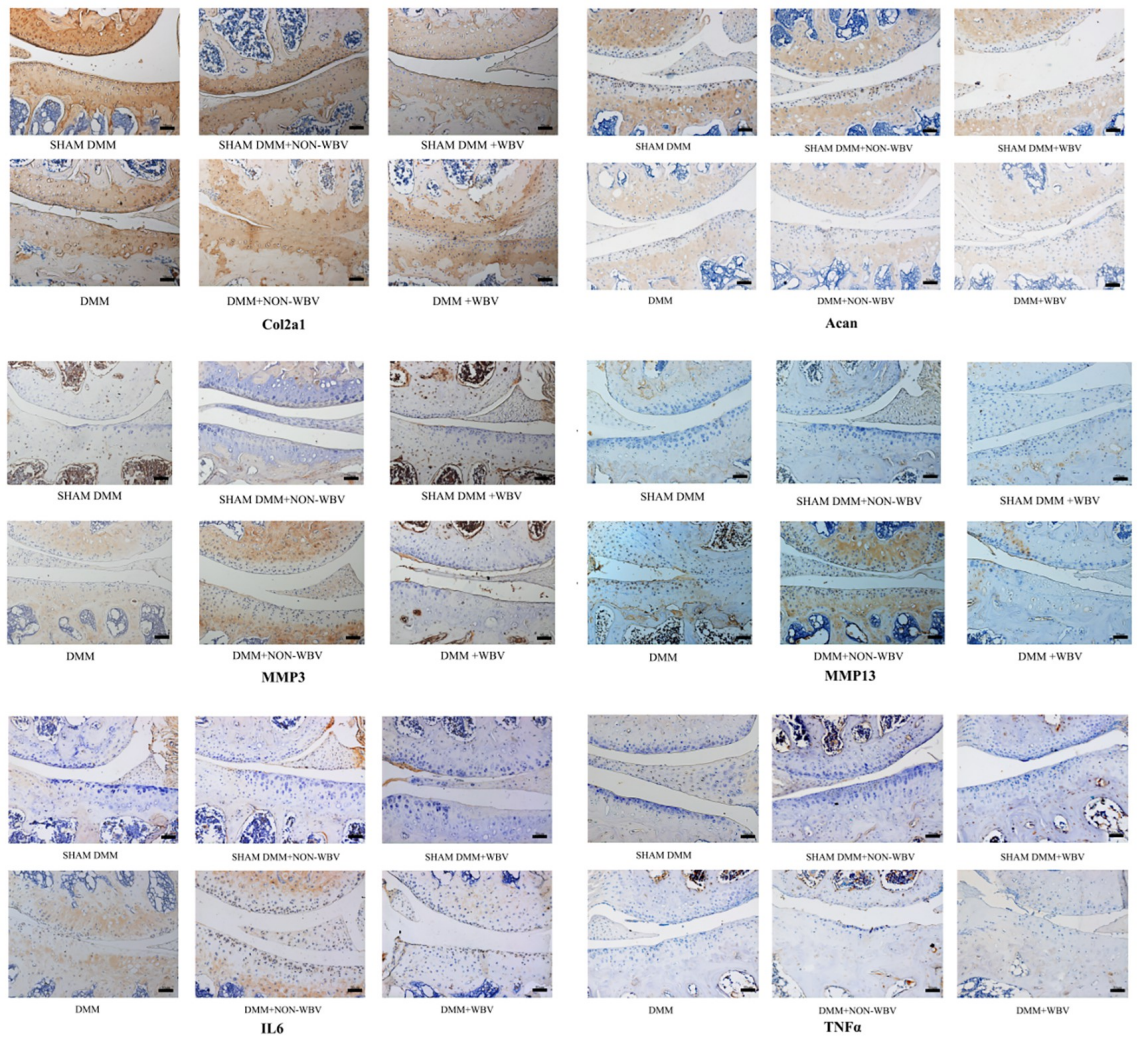
Immunohistochemistry staining for Col2a1, Acan, MMP3, MMP13, IL6 and TNFα in the knee joint cartilage. Compared to the baseline group with sham DMM, the group that underwent DMM surgery exhibited an obvious decrease in Acan and Col2a1, and increase in MMP3, MMP13, IL6 and TNFα. The mouse model of OA eight weeks after surgery showed a further increase in MMP3, MMP13, and an obivious decrease in Acan, Col2a1, compared with those of the baseline OA group. All the proteins detected had a significant decrease in the group DMM+WBV compared with the DMM+NON-WBV group. (n = 6).

We further analyzed protein expressions by immunohistochemistry (Fig 5). The expression of Col2a1 and Acan was significantly decreased in the DMM group than in the SHAM DMM group, while MMP3, MMP13, IL6 and TNFα were increased. The DMM+NON-WBV group showed a further increase in MMP3, MMP13, IL6 and TNFα, and an obvious decrease in Acan, Col2a1, compared with DMM group. All the proteins detected had a significant decrease in the group DMM+WBV compared with the DMM+NON-WBV group, and these results were consistent with the genes detected. There was no obvious difference among the groups with sham DMM, whether WBV was employed or not.

## Discussion

In contrast to our hypothesis that exposure to WBV resulted in cartilage degeneration, healthy joints did not exhibit significant side effects, as compared to those of the matched controls and the baseline group. Further, the combined analysis of all subjects both animals with OA and controls, showed that WBV did not result in obvious changes in subchondral bone.

Mechanics play a crucial role in OA disease progression [4], as mechanical stimulation may be the reason why WBV is popular in OA therapy. WBV could improve the function and muscle strength in the clinical without inducing any adverse effects [10, 17, 21, 22]. However, based on the histological findings, our study demonstrated that WBV resulted in a deterioration of knee joint cartilage in the mice with OA, as shown by the obvious increase in OARSI scores. This finding is also supported by the RT-PCR analysis of anabolism, showing decreased gene expression of Acan and Col2a1, the most important components of cartilage. This result was similar to that of Pamon T [36] and McCann MR [34], one study examined obese mice, exposed to WBV(90 Hz, 0.2 g, 30 min/day, 5 days/week, for 6 weeks), and the other employed CD-1 normal mice for WBV (45 Hz, 0.3 g; 30 min/day, 5 days/week, for 4 weeks and8 weeks). Compared with these studies carried out on normal animals, our report(10 Hz, 0.73 g, 20 min/day, 5 days/week, for 4 weeks)was similar to a study [39] of a rat model of OA treated with WBV (35 Hz, 0.3 g, 20 min/day, 5 days/week, for 6, 12 and 18 weeks). These results suggest that WBV could reduce anabolic genes (Acan and Col2a1) and enhance cartilage degradation. However, catabolism (MMP3 and MMP13) was also depressed, after WBV, as MMP3 and MMP13 are the major collagen-degrading collagenase in cartilage [48, 49]. Anabolism and catabolism are coupled and balanced in healthy joint cartilage, and an imbalance of these processes is typical of OA [50]. Although, anabolic and catabolic components were both decreased, following WBV, the degree of reduction varied, which maybe the reason for cartilage degradation. Compared to NON-WBV group, both anabolic and catabolic decreased too, in the left knees without surgery when WBV employed, which is meaningful. Although morphologic effect on normal joint was not observed, whether WBV has potential damage to normal people deserves further study. WBV also significantly decreased inflammatory cytokines (TNFα and IL6). The change in inflammation in cartilage with OA from the group exposed to WBV is notable, as an increasing number of studies have considered OA to be a low-grade inflammatory disease and the depression of inflammation could relieve OA [51, 52]. Recent studies have revealed that IL6 is a crucial inflammatory factor in OA pathophysiology and that inhibition of IL6 protects against OA [53, 54]. Studies have indicated that TNFα could increase matrix MMPs to degenerate cartilage [55] and cause chondrocyte apoptosis [56]. Researchers have observed that dynamic strain could protect cartilage via inhibiting TNFα and IL6, and could accentuate degradation as well, as shown by the strain threshold [57]. Additionally, Pamon T [36] found that WBV (90 Hz, 0.2 g, 30 min/day, 5 days/week, for 6 weeks) could increase cartilage thickness in obese C57BL/6J mice, while Qin, J [39] showed that WBV(35 Hz, 0.3 g, 20 min/day, 5 days/week, for 6, 12 and 18 weeks)accelerated cartilage degeneration in a rat model of OA. Our latest work [37] (5 Hz, 0.3 g, 20 min/day, 5 days/week, for 4 weeks) showed that WBV could delay cartilage degeneration. Another previous study found local dynamic knee loading employed (5 Hz, 1 N peak to peak, 6 min/day, for 4 weeks) could repair cartilage in a mouse model of OA [58]. Therefore, based on these results and the current findings, researchers should study the effects of specific parameters of WBV, including mechanical stress, on various species. Our work is limited in that the gene expression we detected was from the whole knee joint not just cartilage, which also contains subchondral bone, synovium, ligament and other soft tissues around the joint. Therefore, the result is not sufficiently robust and specific. However, it is difficult to dissect cartilage or synovium free of all soft tissue in mouse knee joints.

Knee joint is a loading joint and its function is mechanical by nature and meanwhile articular cartilage is subjected to loading cycles and mechanical loading plays an important role in regulating development [59]. Biomechanical instability is the prevalent factor involved in the development of posttraumatic osteoarthritis [42] and subchondral bone remodeling is one of another most important characteristics of OA [5], accompanying by the loss of subchondral bone in the early stage, followed by slow turnover leading to sclerosis in the late stage [60]. Drugs cannot reverse the dysfunction caused by progression of OA, and sclerosis in subchondral bone is permanent [5, 60]. Therefore, delaying subchondral bone remodeling may prevent OA. Previous studies have reported that mechanical loading-induced attenuation of sclerostin could increase subchondral bone associated with late stage OA in mice [61] and intermittent mechanical loading induced subchondral bone thickening [62]. The DMM model represented a progression from mild-to-moderate to moderate OA and recommended by OARSI as a gold standard for posttraumatic OA mice experimental study. What we mainly wanted to observe the effect of WBV was mild-to-moderate OA. So, 4 weeks after DMM was chosed. Then, we detected changes in the subchondral bone following WBV via micro-CT. To minimize subjective selection differences and manual measurement errors, we defined the ROI as from the tibial growth plate to the tibial plateau. In this study, we did not obtain obvious evidence in subchondral bone remodeling. However, we found an increasing tendency of Tb. N and a decreasing tendency of TS without significant changes in the OA model exposed to WBV, except BS/BV, which was similar to a study in rats by Qin J [39]. If we consider this in detail, the change of BS/BV mainly lied in increasing of SHAM DMM+WBV group, not decreasing of DMM+WBV group, which really need to investigate deeply, for the increased BS/BV indicating decreased bone strength. In contrast, Junbo W [38] found that low-magnitude WBV (10Hz/20Hz/30Hz/40Hz, 0.3 g, 20 min/day, 5 days/week, for 8 weeks) could prevent loss of trabeculae and increase bone turnover in a rabbit model of OA. Zheng W’s study (5 Hz, 1 N peak to peak, 6 min/day, for 4 weeks) demonstrated that local knee loading could restore cartilage by regulating subchondral bone remodeling via Wnt/β-catenin signaling [58] and our previous finding (5 Hz, 0.3 g, 20 min/day, 5 days/week, last for 4 weeks) also found that WBV could preserve subchondral trabecular bone microarchitecture [37]. Although there was no significant difference in this study, the trend is notable because if WBV could reverse the loss of subchondral bone in early OA, it could delay the progression of this disease. However, it is very interesting that 5HZ WBV could delay cartilage degeneration in our previous study [37], while 10HZ WBV could exacerbate cartilage degeneration in this current study. We assume that frequency maybe the key point for WBV prescription, for different frequency with the opposite biomechanical effects. Therefore, researchers should investigate whether changing the duration or other parameters of WBV results in the same findings.

Joint hypertrophy induced by inflammation of the synovial lining is a notable characteristic sign of OA, that can precede cartilage damage [46, 47] and is also the main reason for pain [63]. Hugle, T [46] concluded that low grade inflammation of synovial tissue is involved in bone remodeling and MRI could be applied to assess both synovitis and the subchondral bone in early OA. We tried to detect joint hypertrophy in general via Vernier caliper, and found no changes. However, the hypertrophic joints of mice with OA exposed to WBV showed a trend of abatement. This alleviation of cartilage swelling may be the reason BS/BV decreased following WBV. The change was not significant, which may be caused by the insufficient detection accuracy of the Vernier caliper. This effect is possibly because WBV could decrease the biomarkers TNFα and IL6 to alleviate inflammation. Simao, A. P [64] also found that WBV (varied from 35 to 40Hz, 2.78 to 3.26g, 20 min/day, 3 days/week, for 12 weeks) could relieve self-perception of pain, and decrease inflammatory markers in elderly patients with knee OA. Therefore, Vernier caliper seems not fit for detection of mouse joint hypertrophy.

OA are characterized by accelerated catabolic processes as well as suppression of anabolic processes. Chondrocytes express various extracellular matrix molecules such as type II collagen and sulfated proteoglycans, which are essential components of chondrocyte anabolism to maintain cartilage homeostasis [65]. Upregulation of matrix-degrading enzymes such as matrix metalloproteinases (MMPs) can lead to cartilage destruction. Low-grade inflammation is an another key mediator of the pathogenesis of osteoarthritis [66]. Inflammatory factors such as IL6, IL1 and TNFα contribute to eliciting imbalance between chondrocyte catabolism and anabolism, then lead to OA [67]. So, we detected anabolism (Col2a1 and Acan) and catabolism (MMP3 and MMP13) biomarkers, and inflammatory cytokines (IL6 and TNFα). Previous work found that WBV could evoke muscular activity, and elevate the metabolic rate, and increase whole and local oxygen uptake [68]. Therefore, as a form of exercise, WBV has been very popular in sports and fitness for decades, with few reported side effects, attracting many people both young and old, and nonobese and obese. However, OA is a disease most commonly found in elderly and obese individuals [1], who may show nonsymptomatic OA. This modality could have negative effects on knee joints if WBV contributed to the degradation of cartilage. Our study did not find any side effects in the normal knees of the mice with OA in the cartilage histologic assessment and subchondral bone, which was similar to the study by Kerr, G. J [35], indicating that WBV would be a safe modality for people with healthy knees. Meanwhile, we also found a significant decrease in Col2a1, Acan and MMP13, and an increase in IL6, in the left normal knee of the mice. Therefore, it is important to study whether this treatment would cause morphological changes of the normal knee joint if the duration of WBV is increased.

The development of osteoarthritis involves loss of cartilage and sclerosis of subchondral bone. Therefore, we detected the subchondral trabecular bone microarchitecture by micro‐CT. We found there were no significant differences in the six groups, in the subchondral BV/TV, BS/BV, Tb.N and TS, except the BS/BV in the DMM+WBV group compared to the SHAM DMM+NON-WBV group, which suggests WBV can enhance subchondral trabecular bone microarchitecture and avoid bone loss. Similar findings were also reported by our previous study [37]. Zhen [69] also found the increase in the size of the subchondral bone could significantly increase stress to articular cartilage. However the relationship between subchondral bone changes and cartilage degradation like a chicken and egg problem, which came first is not clear, need to study deeply. There was a trend of increased BV/TV, Tb.N, and decrease TS, without significant statistical differences, and the reason may be our samples were small or the duration or observation time WBV employed was short.

Notably, the weight of mice with OA was decreased following WBV. However, a previous study [36] (90 Hz, 0.2 g, 30 min/day, 5 days/week, for 6 weeks) which employed normal C57BL/6J mice did not report weight changes, while clinically, Figueroa [70] also found that the weight of young overweight/obese women did not change after WBV training (25–30Hz, 2.83–4.86g, 3 days/week, for 6 weeks). Qin, J [39] found that a higher level of pain was induced in the OA affected limb after WBV training. Therefore, we speculated that WBV application led to cartilage degeneration exacerbating OA pain, which may decrease feeding, and future studies are needed.

In conclusion, WBV(10 Hz, 0.73 g, 20 min/day, 5 days/week, for 4 weeks) results in progressive degeneration of articular cartilage, with few changes in subchondral bone in mice with knee OA. Furthermore, we found that WBV did not have any morphologic effect on normal joints.

## Supporting information

S1 File (DOCX)
