# Supplementary material for: Low-frequency whole-body vibration can enhance cartilage degradation with slight changes in subchondral bone in mice with knee osteoarthritis and does not have any morphologic effect on normal joints
Source: PLoS One. 2023 Aug 17;18(8):e0270074. doi: 10.1371/journal.pone.0270074 (PMC10434961; doi:10.1371/journal.pone.0270074)
Supplement: S1 File — (DOCX) [file pone.0270074.s001.docx]

Supplemental files

Table.S1 Data of OARSI score

| Group | Each sample Data of OARSI score | | | | | |
| --- | --- | --- | --- | --- | --- | --- |
|  | n1 | n2 | n3 | n4 | n5 | n6 |
| SHAM DMM | 0.5 | 0 | 0 | 0 | 0 | 1 |
| DMM | 2 | 4 | 2 | 4 | 3 | 3 |
| SHAM DMM+NON-WBV | 0 | 1 | 1 | 0.5 | 0.5 | 0 |
| DMM+ NON-WBV | 4 | 3 | 4 | 3 | 3 | 4 |
| SHAM DMM+WBV | 0 | 1 | 0.5 | 0 | 0 | 0.5 |
| DMM+WBV | 6 | 2 | 6 | 5 | 4 | 5 |

Knee joints were scored using the OARSI scoring system to quantify the degree of joint degeneration, and the OARSI scores were presented corresponding to the medial articular surface(n=6). The tibial plateau quadrants of the knee joint were scored by two independent blinded observers according the mouse recommendations of OARSI. OA severity is expressed by the mean score.

Table.S2 Micro-CT data of tibial subchondral bone microarchitecture

|  | SHAM DMM | DMM | SHAM DMM+NON-WBV | DMM+ NON-WBV | SHAM DMM+WBV | DMM+WBV |
| --- | --- | --- | --- | --- | --- | --- |
| BV/TV | 0.32±0.0361 | 0.36±0.0992 | 0.30±0.0396 | 0.34±0.0687 | 0.30±0.0411 | 0.36±0.0597 |
| BS/BV | 58.92±4.7201 | 58.30±6.8868 | 56.68±7.0651 | 55.06±7.213 | 62.71±8.8632 | 55.14±1.0479* |
| TN | 9.44±0.7968 | 10.41±1.7355 | 8.36±0.6341 | 9.20±0.5477 | 9.20±0.1987 | 9.81±1.7024 |
| TS | 0.0724±0.0092 | 0.0635±0.0192 | 0.0845±0.0090 | 0.0721±0.0115 | 0.0762±0.0042 | 0.0680±0.0168 |

Morphometric analysis of the subchondral bone in all groups, with or without WBV, shows no significant change in the bone microstructure parameters, including bone volume to tissue volume ratio (BV/TV), bone surface area to bone volume ratio (BS/BV), trabecular number (Tb. N) and trabecular thickness separation (TS) in all the groups, except OA group exposed to WBV compared to the matched control group with sham DMM.(n=6). Data are expressed as the mean±SD. *compare with SHAM DMM+ WBV group (P=0.043).

Table.S3 Data of relative mRNA expression levels of Acan, Col2a1, MMP3, MMP13, TNFα and IL6

| Group | Col2a1 | Acan | MMP3 | MMP13, | IL6 | TNFα |
| --- | --- | --- | --- | --- | --- | --- |
| SHAM DMM | 1.69±0.25 | 1.83±0.41 | 0.03±0.01 | 1.06±0.33 | 0.06±0.05 | 0.09±0.00 |
| DMM | 3.99±0.28**^﹡^** | 3.86±0.281**^﹡^** | 3.01±0.33**^﹡^** | 2.44±0.24**^﹡^** | 0.21±0.05**^﹡^** | 0.11±0.06 |
| SHAM DMM+NON-WBV | 1.08±0.16 | 1.71±0.49 | 0.03±0.02 | 1.06±0.18 | 0.04±0.04 | 0.12±0.02 |
| DMM+ NON-WBV | 2.35±0.33**^＃^** | 2.87±0.24**^＃^** | 3.68±0.12**^＃﹡^** | 3.19±0.19**^＃﹡^** | 1.37±0.22**^＃﹡^** | 0.71±0.33**^＃﹡^** |
| SHAM DMM+WBV | 0.49±0.28**^※^** | 0.80±0.38**^※^** | 0.03±0.02 | 0.63±0.15**^※^** | 0.26±0.05**^※^** | 0.10±0.04 |
| DMM+WBV | 0.58±0.36**^§^** | 0.78±0.41**^§^** | 2.30±0.34**^§^** | 0.76±0.15**^§^** | 0.34±0.11**^§^** | 0.09±0.02**^§^** |

Relative mRNA expression levels of Acan, Col2a1, MMP3, MMP13, TNFα and IL6 in the knee joint cartilage.﹡compare with SHAM DMM group (P<0.0001), ＃compare with DMM group (P<0.0001), §compare with DMM+ NON-WBV group (P<0.0001), ※compare with SHAM DMM+ NON-WBV group (P<0.0001)
